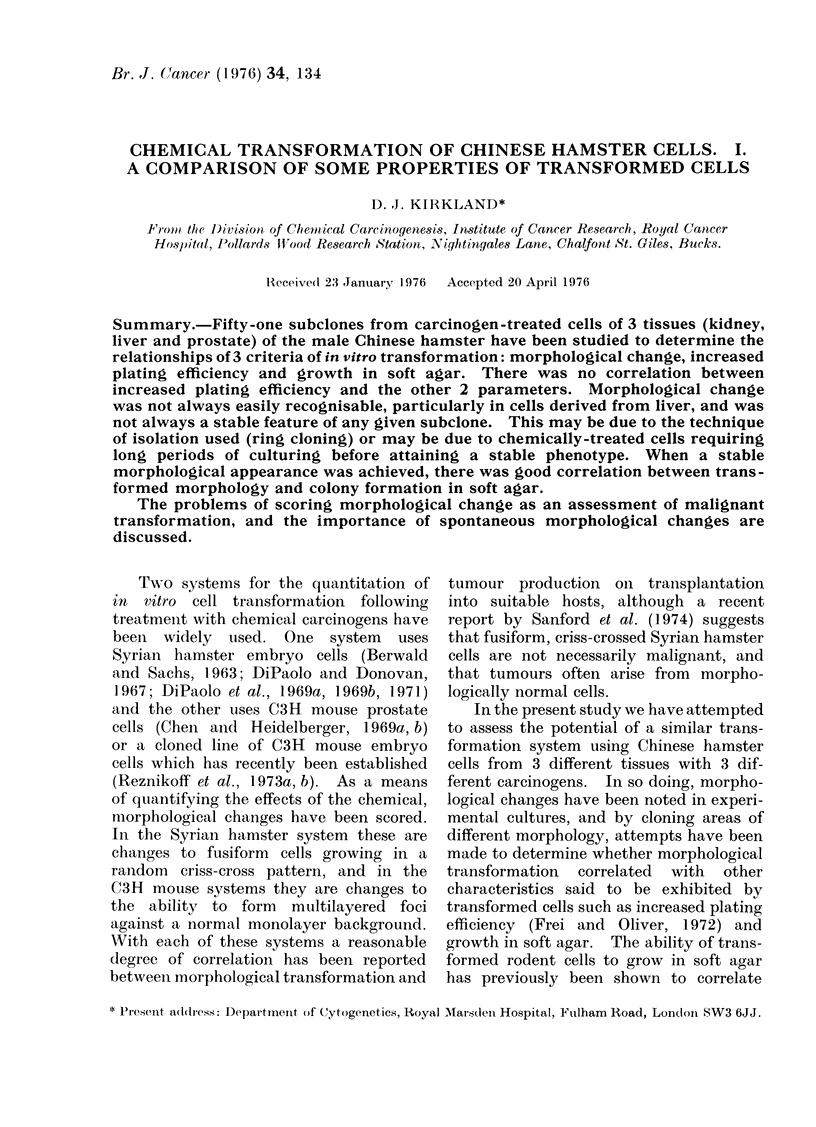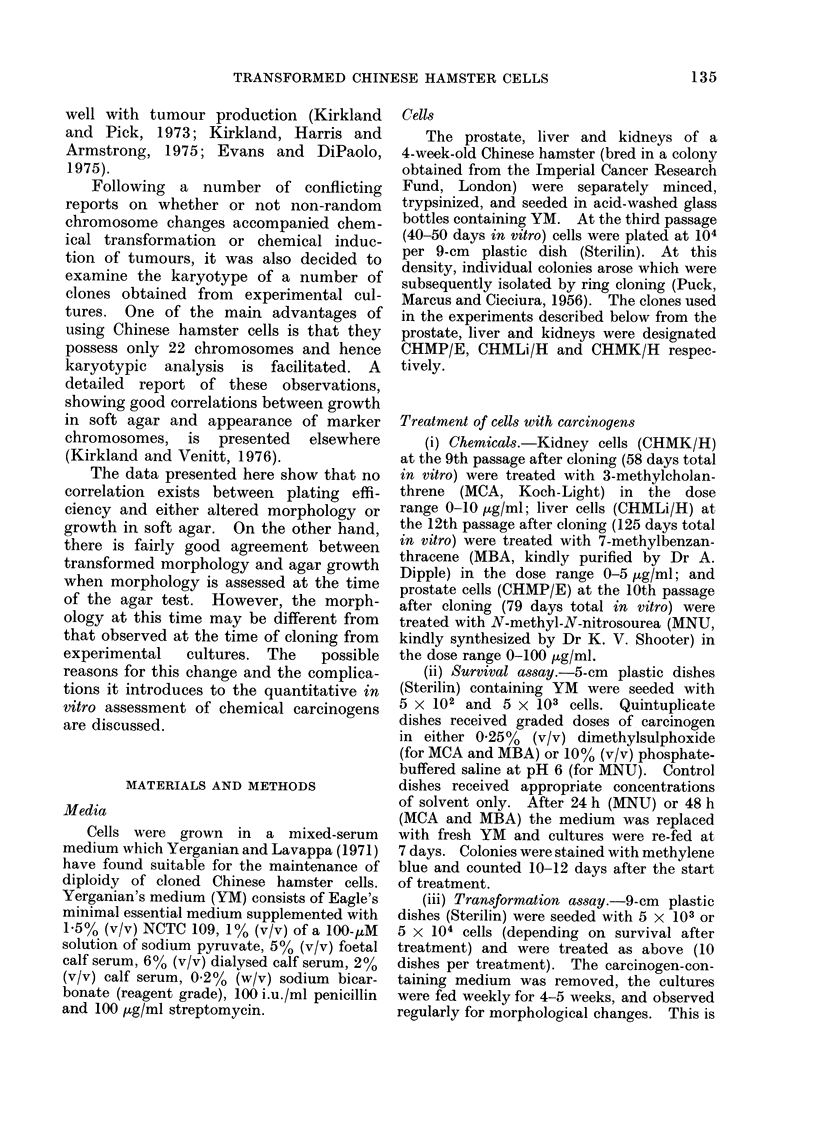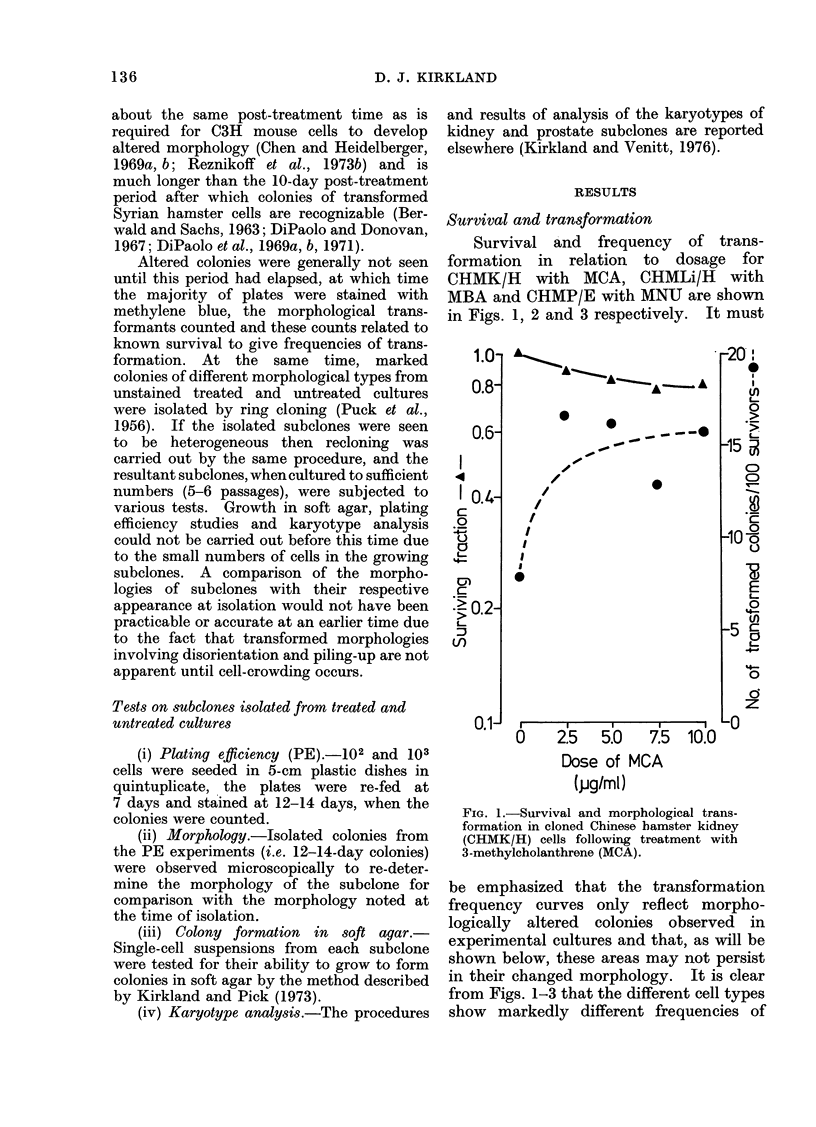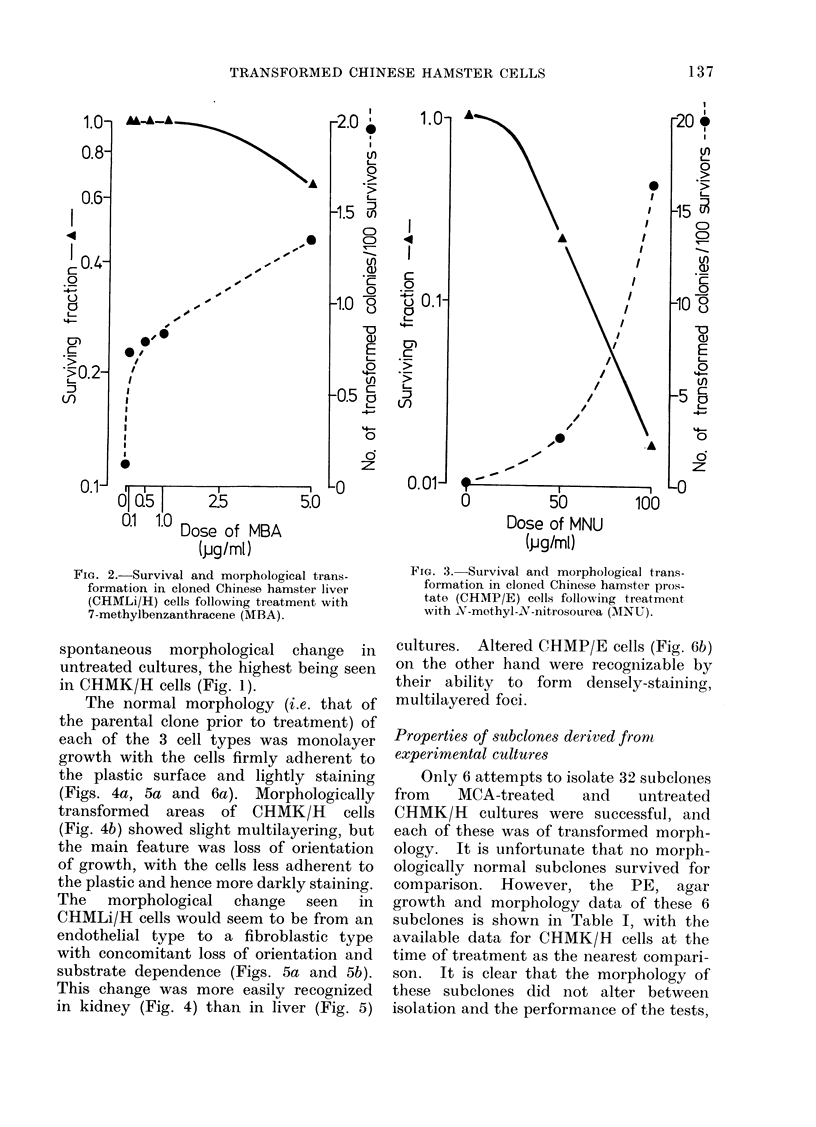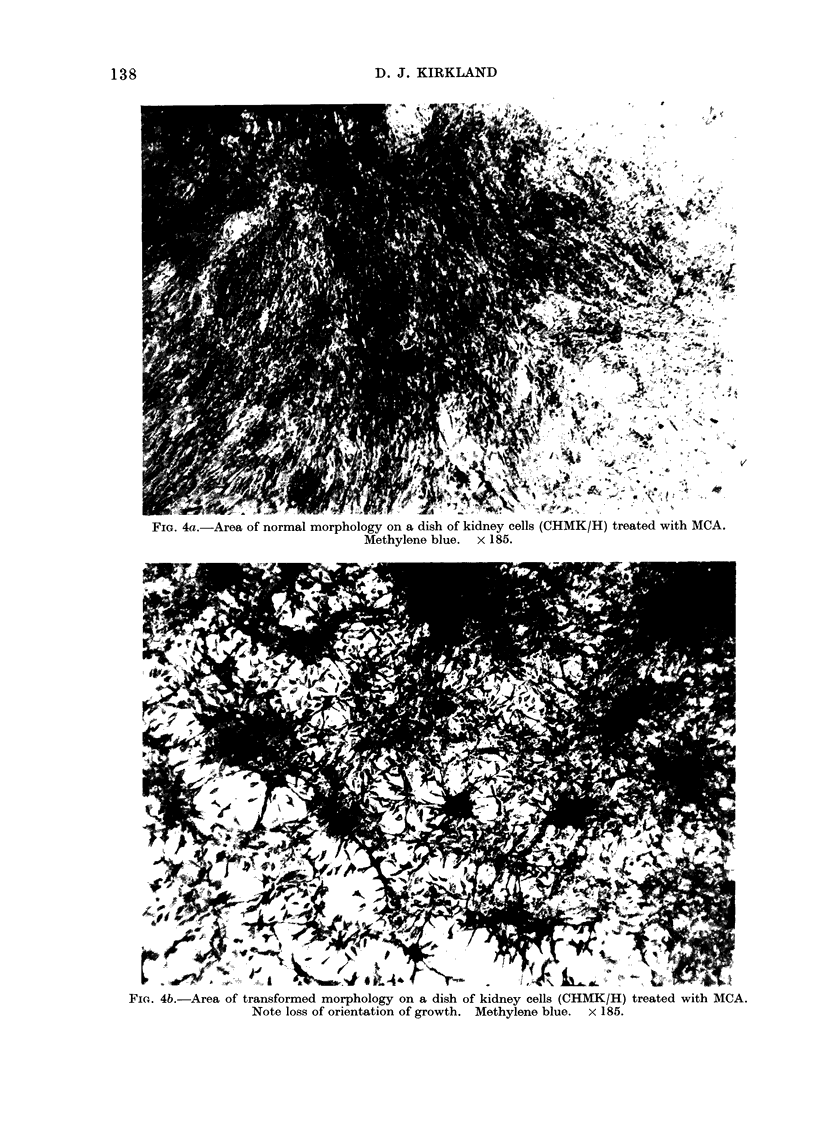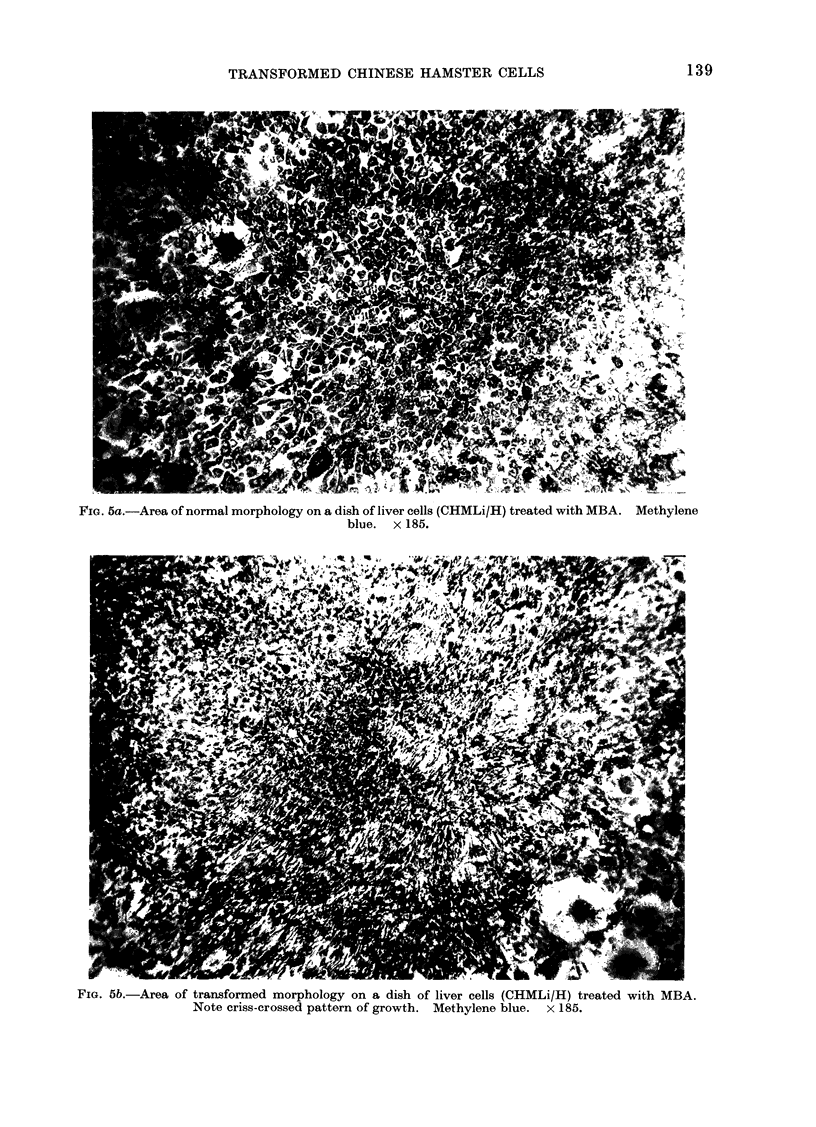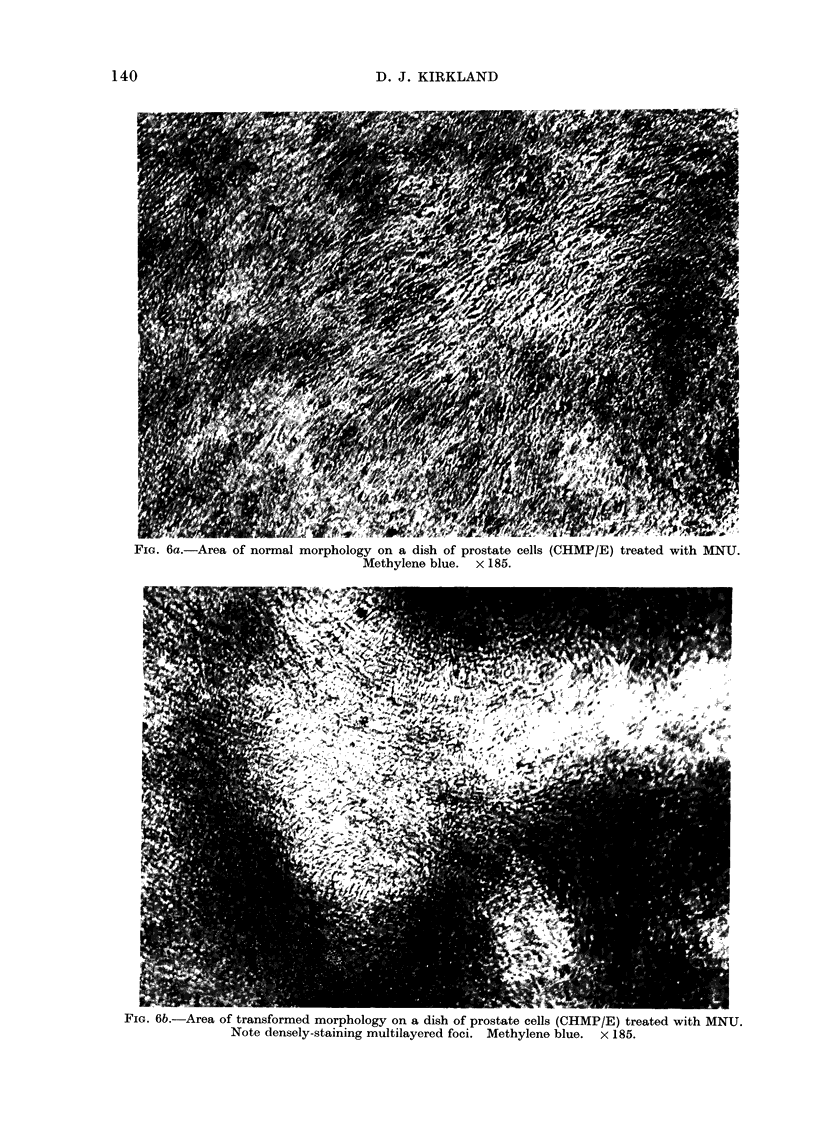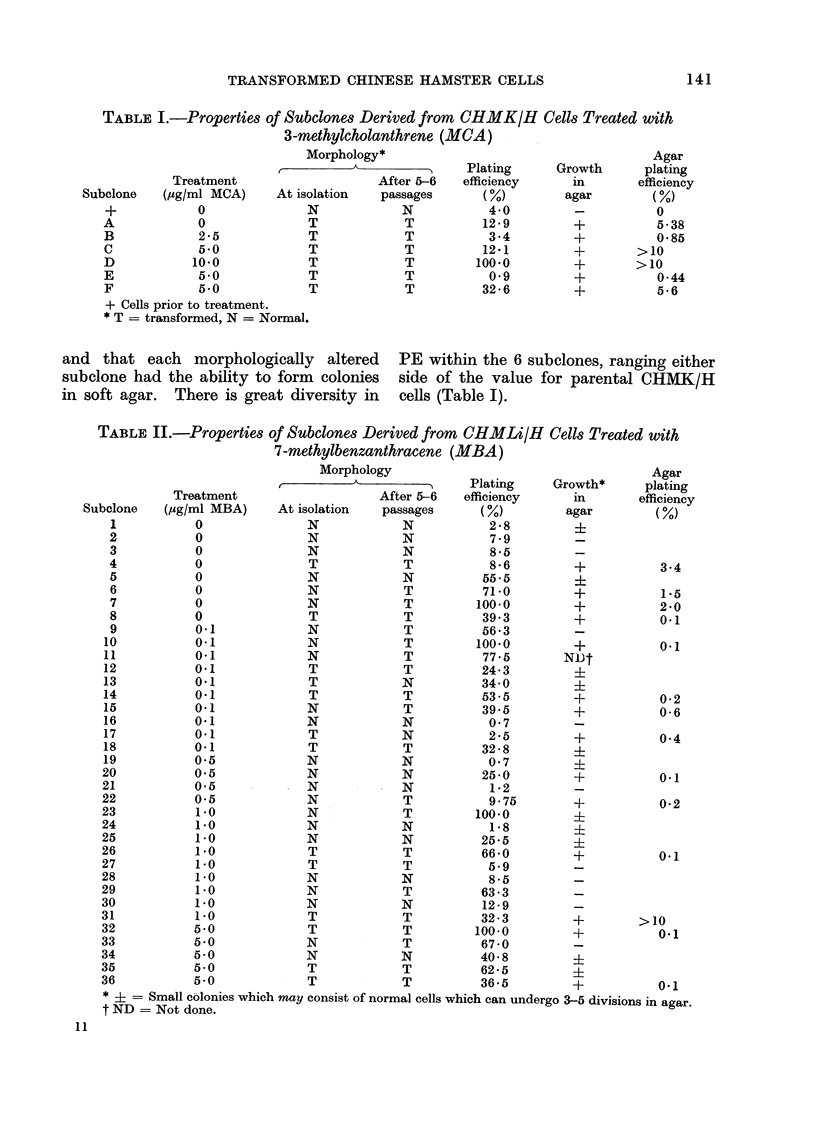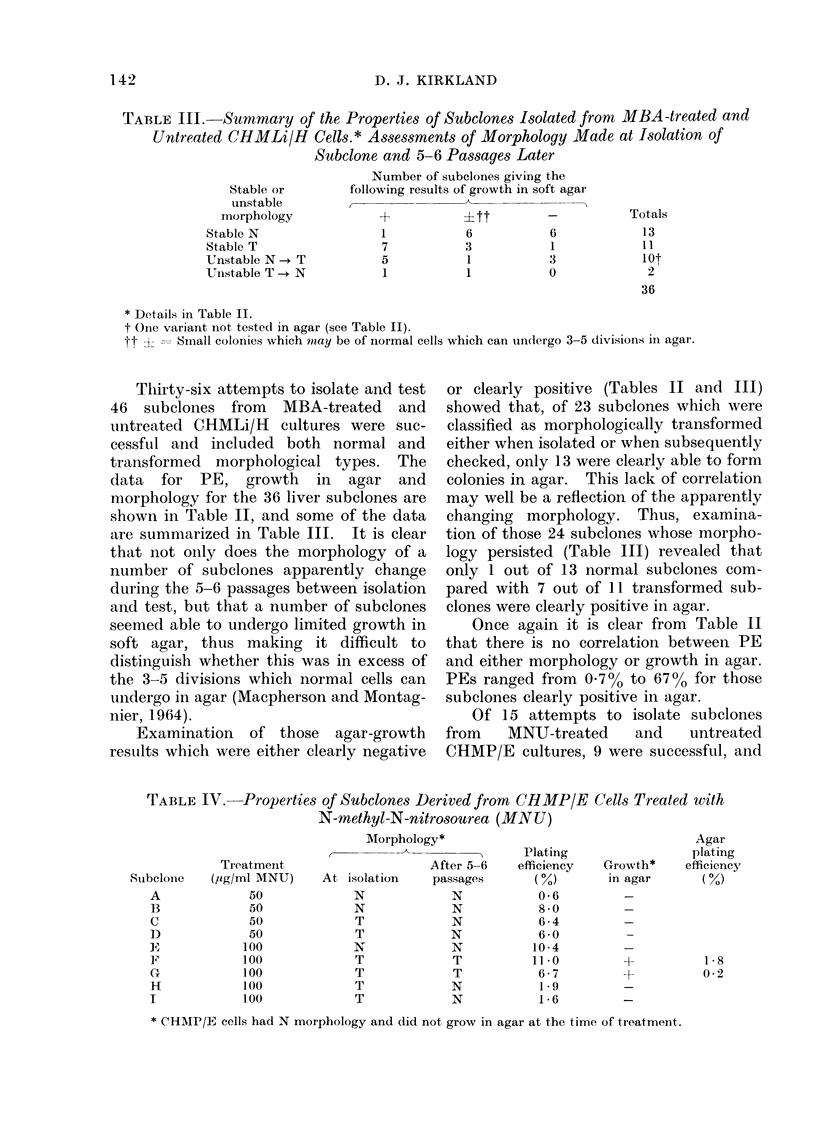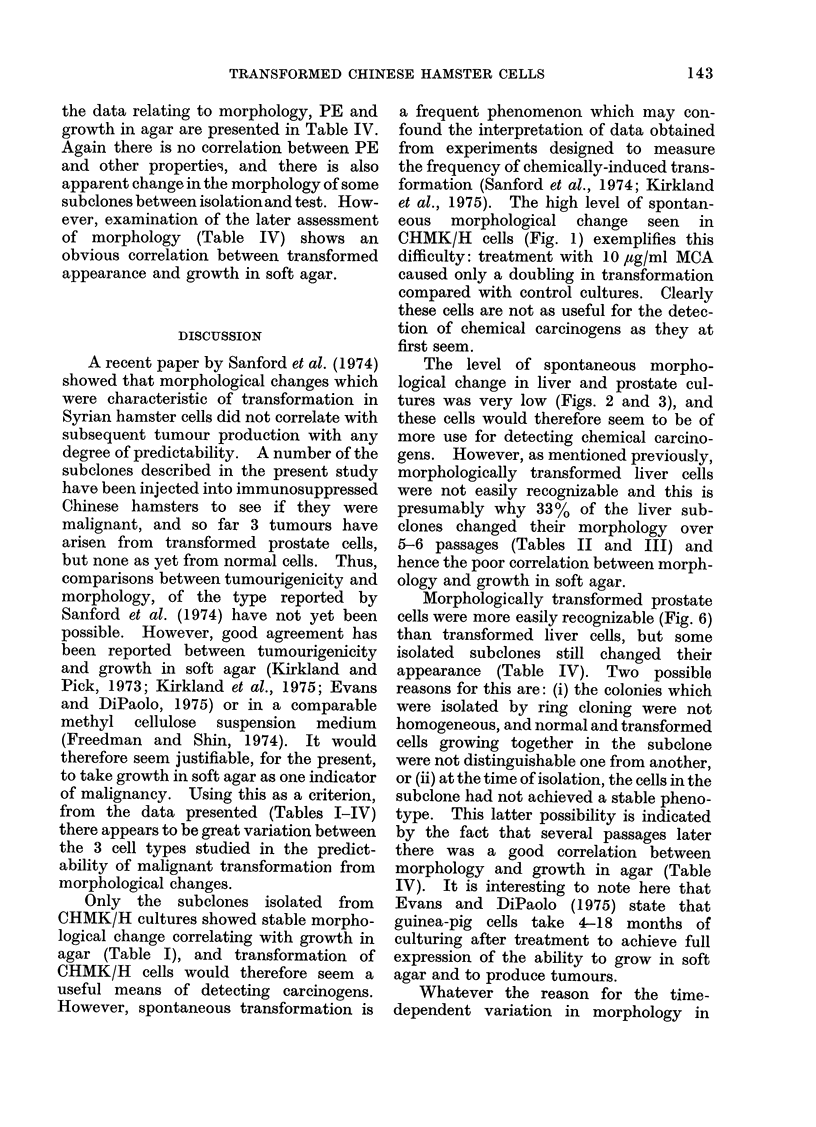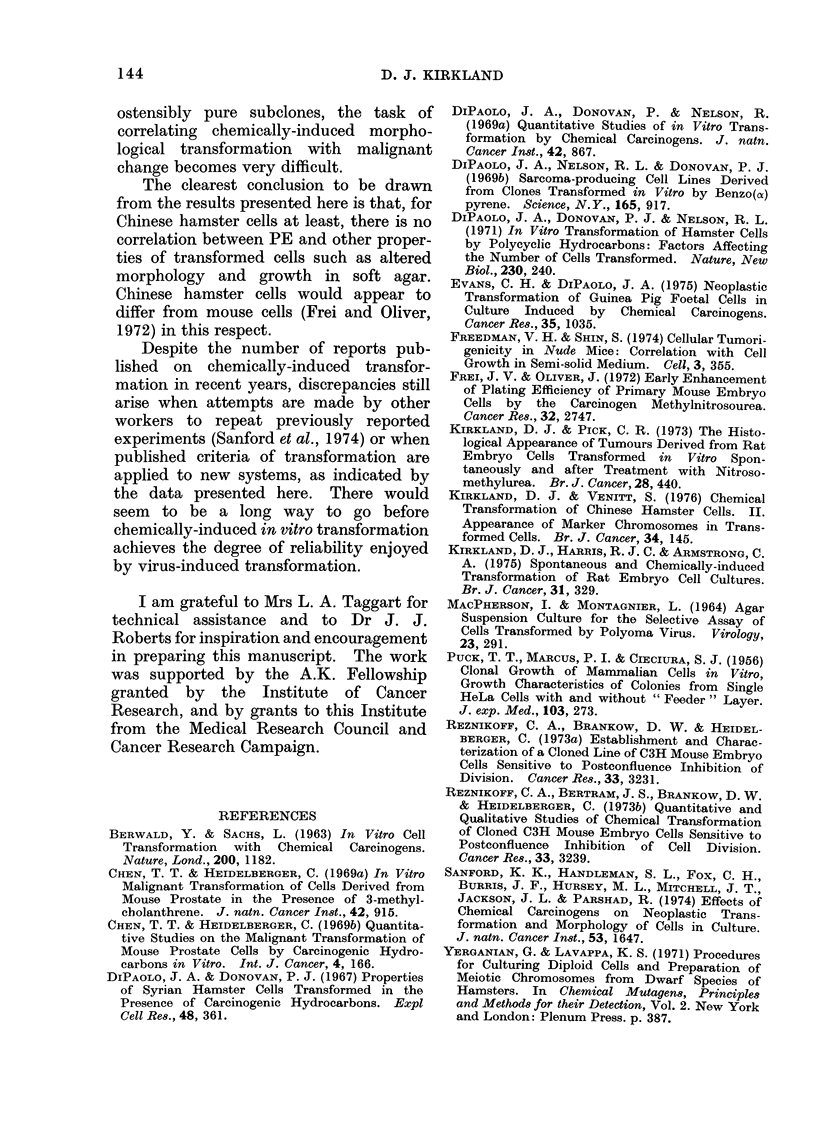# Chemical transformation of Chinese hamster cells. I. A comparison of some properties of transformed cells.

**DOI:** 10.1038/bjc.1976.135

**Published:** 1976-08

**Authors:** D. J. Kirkland

## Abstract

**Images:**


*Br. J. Cancer* (1976) **34**, 134

### CHEMICAL TRANSFORMATION OF CHINESE HAMSTER CELLS. I. A COMPARISON OF SOME PROPERTIES OF TRANSFORMED CELLS

D. J. KIRKLAND*

*From the Division of Chemical Carcinogenesis, Institute of Cancer Research, Royal Cancer Hospital, Pollards Wood Research Station, Nightingales Lane, Chalfont St. Giles, Bucks.*

Received 23 January 1976 Accepted 20 April 1976

**Summary.—Fifty-one subclones from carcinogen-treated cells of 3 tissues (kidney, liver and prostate) of the male Chinese hamster have been studied to determine the relationships of 3 criteria of *in vitro* transformation: morphological change, increased plating efficiency and growth in soft agar. There was no correlation between increased plating efficiency and the other 2 parameters. Morphological change was not always easily recognisable, particularly in cells derived from liver, and was not always a stable feature of any given subclone. This may be due to the technique of isolation used (ring cloning) or may be due to chemically-treated cells requiring long periods of culturing before attaining a stable phenotype. When a stable morphological appearance was achieved, there was good correlation between transformed morphology and colony formation in soft agar.**

**The problems of scoring morphological change as an assessment of malignant transformation, and the importance of spontaneous morphological changes are discussed.**

Two systems for the quantitation of *in vitro* cell transformation following treatment with chemical carcinogens have been widely used. One system uses Syrian hamster embryo cells (Berwald and Sachs, 1963; DiPaolo and Donovan, 1967; DiPaolo *et al.*, 1969*a*, 1969*b*, 1971) and the other uses C3H mouse prostate cells (Chen and Heidelberger, 1969*a*, *b*) or a cloned line of C3H mouse embryo cells which has recently been established (Reznikoff *et al.*, 1973*a*, *b*). As a means of quantifying the effects of the chemical, morphological changes have been scored. In the Syrian hamster system these are changes to fusiform cells growing in a random criss-cross pattern, and in the C3H mouse systems they are changes to the ability to form multilayered foci against a normal monolayer background. With each of these systems a reasonable degree of correlation has been reported between morphological transformation and tumour production on transplantation into suitable hosts, although a recent report by Sanford *et al.* (1974) suggests that fusiform, criss-crossed Syrian hamster cells are not necessarily malignant, and that tumours often arise from morphologically normal cells.

In the present study we have attempted to assess the potential of a similar transformation system using Chinese hamster cells from 3 different tissues with 3 different carcinogens. In so doing, morphological changes have been noted in experimental cultures, and by cloning areas of different morphology, attempts have been made to determine whether morphological transformation correlated with other characteristics said to be exhibited by transformed cells such as increased plating efficiency (Frei and Oliver, 1972) and growth in soft agar. The ability of transformed rodent cells to grow in soft agar has previously been shown to correlate

* Present address: Department of Cytogenetics, Royal Marsden Hospital, Fulham Road, London SW3 6JJ.

well with tumour production (Kirkland and Pick, 1973; Kirkland, Harris and Armstrong, 1975; Evans and DiPaolo, 1975).

Following a number of conflicting reports on whether or not non-random chromosome changes accompanied chemical transformation or chemical induction of tumours, it was also decided to examine the karyotype of a number of clones obtained from experimental cultures. One of the main advantages of using Chinese hamster cells is that they possess only 22 chromosomes and hence karyotypic analysis is facilitated. A detailed report of these observations, showing good correlations between growth in soft agar and appearance of marker chromosomes, is presented elsewhere (Kirkland and Venitt, 1976).

The data presented here show that no correlation exists between plating efficiency and either altered morphology or growth in soft agar. On the other hand, there is fairly good agreement between transformed morphology and agar growth when morphology is assessed at the time of the agar test. However, the morphology at this time may be different from that observed at the time of cloning from experimental cultures. The possible reasons for this change and the complications it introduces to the quantitative *in vitro* assessment of chemical carcinogens are discussed.

### MATERIALS AND METHODS

### *Media*

Cells were grown in a mixed-serum medium which Yerganian and Lavappa (1971) have found suitable for the maintenance of diploidy of cloned Chinese hamster cells. Yerganian's medium (YM) consists of Eagle's minimal essential medium supplemented with 1·5% (v/v) NCTC 109, 1% (v/v) of a 100-$\mu$M solution of sodium pyruvate, 5% (v/v) foetal calf serum, 6% (v/v) dialysed calf serum, 2% (v/v) calf serum, 0·2% (w/v) sodium bicarbonate (reagent grade), 100 i.u./ml penicillin and 100 $\mu$g/ml streptomycin.

### *Cells*

The prostate, liver and kidneys of a 4-week-old Chinese hamster (bred in a colony obtained from the Imperial Cancer Research Fund, London) were separately minced, trypsinized, and seeded in acid-washed glass bottles containing YM. At the third passage (40–50 days *in vitro*) cells were plated at $10^4$ per 9-cm plastic dish (Sterilin). At this density, individual colonies arose which were subsequently isolated by ring cloning (Puck, Marcus and Cieciura, 1956). The clones used in the experiments described below from the prostate, liver and kidneys were designated CHMP/E, CHMLi/H and CHMK/H respectively.

### *Treatment of cells with carcinogens*

(i) *Chemicals.*—Kidney cells (CHMK/H) at the 9th passage after cloning (58 days total *in vitro*) were treated with 3-methylcholanthrene (MCA, Koch-Light) in the dose range 0–10 $\mu$g/ml; liver cells (CHMLi/H) at the 12th passage after cloning (125 days total *in vitro*) were treated with 7-methylbenzanthracene (MBA, kindly purified by Dr A. Dipple) in the dose range 0–5 $\mu$g/ml; and prostate cells (CHMP/E) at the 10th passage after cloning (79 days total *in vitro*) were treated with *N*-methyl-*N*-nitrosourea (MNU, kindly synthesized by Dr K. V. Shooter) in the dose range 0–100 $\mu$g/ml.

(ii) *Survival assay.*—5-cm plastic dishes (Sterilin) containing YM were seeded with $5 \times 10^2$ and $5 \times 10^3$ cells. Quintuplicate dishes received graded doses of carcinogen in either 0·25% (v/v) dimethylsulphoxide (for MCA and MBA) or 10% (v/v) phosphate-buffered saline at pH 6 (for MNU). Control dishes received appropriate concentrations of solvent only. After 24 h (MNU) or 48 h (MCA and MBA) the medium was replaced with fresh YM and cultures were re-fed at 7 days. Colonies were stained with methylene blue and counted 10–12 days after the start of treatment.

(iii) *Transformation assay.*—9-cm plastic dishes (Sterilin) were seeded with $5 \times 10^3$ or $5 \times 10^4$ cells (depending on survival after treatment) and were treated as above (10 dishes per treatment). The carcinogen-containing medium was removed, the cultures were fed weekly for 4–5 weeks, and observed regularly for morphological changes. This is about the same post-treatment time as is required for C3H mouse cells to develop altered morphology (Chen and Heidelberger, 1969*a*, *b*; Reznikoff *et al.*, 1973*b*) and is much longer than the 10-day post-treatment period after which colonies of transformed Syrian hamster cells are recognizable (Berwald and Sachs, 1963; DiPaolo and Donovan, 1967; DiPaolo *et al.*, 1969*a*, *b*, 1971).

Altered colonies were generally not seen until this period had elapsed, at which time the majority of plates were stained with methylene blue, the morphological transformants counted and these counts related to known survival to give frequencies of transformation. At the same time, marked colonies of different morphological types from unstained treated and untreated cultures were isolated by ring cloning (Puck *et al.*, 1956). If the isolated subclones were seen to be heterogeneous then recloning was carried out by the same procedure, and the resultant subclones, when cultured to sufficient numbers (5–6 passages), were subjected to various tests. Growth in soft agar, plating efficiency studies and karyotype analysis could not be carried out before this time due to the small numbers of cells in the growing subclones. A comparison of the morphologies of subclones with their respective appearance at isolation would not have been practicable or accurate at an earlier time due to the fact that transformed morphologies involving disorientation and piling-up are not apparent until cell-crowding occurs.

*Tests on subclones isolated from treated and untreated cultures*

(i) *Plating efficiency* (PE).—$10^2$ and $10^3$ cells were seeded in 5-cm plastic dishes in quintuplicate, the plates were re-fed at 7 days and stained at 12–14 days, when the colonies were counted.

(ii) *Morphology*.—Isolated colonies from the PE experiments (*i.e.* 12–14-day colonies) were observed microscopically to re-determine the morphology of the subclone for comparison with the morphology noted at the time of isolation.

(iii) *Colony formation in soft agar*.—Single-cell suspensions from each subclone were tested for their ability to grow to form colonies in soft agar by the method described by Kirkland and Pick (1973).

(iv) *Karyotype analysis*.—The procedures and results of analysis of the karyotypes of kidney and prostate subclones are reported elsewhere (Kirkland and Venitt, 1976).

### RESULTS

*Survival and transformation*

Survival and frequency of transformation in relation to dosage for CHMK/H with MCA, CHMLi/H with MBA and CHMP/E with MNU are shown in Figs. 1, 2 and 3 respectively. It must

FIG. 1.—Survival and morphological transformation in cloned Chinese hamster kidney (CHMK/H) cells following treatment with 3-methylcholanthrene (MCA).

be emphasized that the transformation frequency curves only reflect morphologically altered colonies observed in experimental cultures and that, as will be shown below, these areas may not persist in their changed morphology. It is clear from Figs. 1–3 that the different cell types show markedly different frequencies of

Fig. 2.—Survival and morphological transformation in cloned Chinese hamster liver (CHMLi/H) cells following treatment with 7-methylbenzanthracene (MBA).

Fig. 3.—Survival and morphological transformation in cloned Chinese hamster prostate (CHMP/E) cells following treatment with *N*-methyl-*N*-nitrosourea (MNU).

spontaneous morphological change in untreated cultures, the highest being seen in CHMK/H cells (Fig. 1).

The normal morphology (*i.e.* that of the parental clone prior to treatment) of each of the 3 cell types was monolayer growth with the cells firmly adherent to the plastic surface and lightly staining (Figs. 4*a*, 5*a* and 6*a*). Morphologically transformed areas of CHMK/H cells (Fig. 4*b*) showed slight multilayering, but the main feature was loss of orientation of growth, with the cells less adherent to the plastic and hence more darkly staining. The morphological change seen in CHMLi/H cells would seem to be from an endothelial type to a fibroblastic type with concomitant loss of orientation and substrate dependence (Figs. 5*a* and 5*b*). This change was more easily recognized in kidney (Fig. 4) than in liver (Fig. 5) cultures. Altered CHMP/E cells (Fig. 6*b*) on the other hand were recognizable by their ability to form densely-staining, multilayered foci.

### *Properties of subclones derived from experimental cultures*

Only 6 attempts to isolate 32 subclones from MCA-treated and untreated CHMK/H cultures were successful, and each of these was of transformed morphology. It is unfortunate that no morphologically normal subclones survived for comparison. However, the PE, agar growth and morphology data of these 6 subclones is shown in Table I, with the available data for CHMK/H cells at the time of treatment as the nearest comparison. It is clear that the morphology of these subclones did not alter between isolation and the performance of the tests,

FIG. 4*a*.—Area of normal morphology on a dish of kidney cells (CHMK/H) treated with MCA. Methylene blue. ×185.

FIG. 4*b*.—Area of transformed morphology on a dish of kidney cells (CHMK/H) treated with MCA. Note loss of orientation of growth. Methylene blue. ×185.

FIG. 5a.—Area of normal morphology on a dish of liver cells (CHMLi/H) treated with MBA. Methylene blue. ×185.

FIG. 5b.—Area of transformed morphology on a dish of liver cells (CHMLi/H) treated with MBA. Note criss-crossed pattern of growth. Methylene blue. ×185.

FIG. 6*a*.—Area of normal morphology on a dish of prostate cells (CHMP/E) treated with MNU. Methylene blue. ×185.

FIG. 6*b*.—Area of transformed morphology on a dish of prostate cells (CHMP/E) treated with MNU. Note densely-staining multilayered foci. Methylene blue. ×185.

TABLE I.—*Properties of Subclones Derived from CHMK/H Cells Treated with 3-methylcholanthrene (MCA)*

| Subclone | Treatment (μg/ml MCA) | Morphology* At isolation | Morphology* After 5–6 passages | Plating efficiency (%) | Growth in agar | Agar plating efficiency (%) |
|---|---|---|---|---|---|---|
| + | 0 | N | N | 4·0 | − | 0 |
| A | 0 | T | T | 12·9 | + | 5·38 |
| B | 2·5 | T | T | 3·4 | + | 0·85 |
| C | 5·0 | T | T | 12·1 | + | >10 |
| D | 10·0 | T | T | 100·0 | + | >10 |
| E | 5·0 | T | T | 0·9 | + | 0·44 |
| F | 5·0 | T | T | 32·6 | + | 5·6 |

\+ Cells prior to treatment.
* T = transformed, N = Normal.

and that each morphologically altered subclone had the ability to form colonies in soft agar. There is great diversity in PE within the 6 subclones, ranging either side of the value for parental CHMK/H cells (Table I).

TABLE II.—*Properties of Subclones Derived from CHMLi/H Cells Treated with 7-methylbenzanthracene (MBA)*

| Subclone | Treatment (μg/ml MBA) | Morphology At isolation | Morphology After 5–6 passages | Plating efficiency (%) | Growth* in agar | Agar plating efficiency (%) |
|---|---|---|---|---|---|---|
| 1 | 0 | N | N | 2·8 | ± | |
| 2 | 0 | N | N | 7·9 | − | |
| 3 | 0 | N | N | 8·5 | − | |
| 4 | 0 | T | T | 8·6 | + | 3·4 |
| 5 | 0 | N | N | 55·5 | ± | |
| 6 | 0 | N | T | 71·0 | + | 1·5 |
| 7 | 0 | N | T | 100·0 | + | 2·0 |
| 8 | 0 | T | T | 39·3 | + | 0·1 |
| 9 | 0·1 | N | T | 56·3 | − | |
| 10 | 0·1 | N | T | 100·0 | + | 0·1 |
| 11 | 0·1 | N | T | 77·5 | ND† | |
| 12 | 0·1 | T | T | 24·3 | ± | |
| 13 | 0·1 | T | N | 34·0 | ± | |
| 14 | 0·1 | T | T | 53·5 | + | 0·2 |
| 15 | 0·1 | N | T | 39·5 | + | 0·6 |
| 16 | 0·1 | N | N | 0·7 | − | |
| 17 | 0·1 | T | N | 2·5 | + | 0·4 |
| 18 | 0·1 | T | T | 32·8 | ± | |
| 19 | 0·5 | N | N | 0·7 | ± | |
| 20 | 0·5 | N | N | 25·0 | + | 0·1 |
| 21 | 0·5 | N | N | 1·2 | − | |
| 22 | 0·5 | N | T | 9·75 | + | 0·2 |
| 23 | 1·0 | N | T | 100·0 | ± | |
| 24 | 1·0 | N | N | 1·8 | ± | |
| 25 | 1·0 | N | N | 25·5 | ± | |
| 26 | 1·0 | T | T | 66·0 | + | 0·1 |
| 27 | 1·0 | T | T | 5·9 | − | |
| 28 | 1·0 | N | N | 8·5 | − | |
| 29 | 1·0 | N | T | 63·3 | − | |
| 30 | 1·0 | N | N | 12·9 | − | |
| 31 | 1·0 | T | T | 32·3 | + | >10 |
| 32 | 5·0 | T | T | 100·0 | + | 0·1 |
| 33 | 5·0 | N | T | 67·0 | − | |
| 34 | 5·0 | N | N | 40·8 | ± | |
| 35 | 5·0 | T | T | 62·5 | ± | |
| 36 | 5·0 | T | T | 36·5 | + | 0·1 |

* ± = Small colonies which *may* consist of normal cells which can undergo 3–5 divisions in agar.
† ND = Not done.

TABLE III.—*Summary of the Properties of Subclones Isolated from MBA-treated and Untreated CHMLi/H Cells.* Assessments of Morphology Made at Isolation of Subclone and 5–6 Passages Later*

| Stable or unstable morphology | Number of subclones giving the following results of growth in soft agar | | | Totals |
|---|---|---|---|---|
| | + | ±†† | − | |
| Stable N | 1 | 6 | 6 | 13 |
| Stable T | 7 | 3 | 1 | 11 |
| Unstable N → T | 5 | 1 | 3 | 10† |
| Unstable T → N | 1 | 1 | 0 | 2 |
| | | | | 36 |

* Details in Table II.
† One variant not tested in agar (see Table II).
†† ± = Small colonies which *may* be of normal cells which can undergo 3–5 divisions in agar.

Thirty-six attempts to isolate and test 46 subclones from MBA-treated and untreated CHMLi/H cultures were successful and included both normal and transformed morphological types. The data for PE, growth in agar and morphology for the 36 liver subclones are shown in Table II, and some of the data are summarized in Table III. It is clear that not only does the morphology of a number of subclones apparently change during the 5–6 passages between isolation and test, but that a number of subclones seemed able to undergo limited growth in soft agar, thus making it difficult to distinguish whether this was in excess of the 3–5 divisions which normal cells can undergo in agar (Macpherson and Montagnier, 1964).

Examination of those agar-growth results which were either clearly negative or clearly positive (Tables II and III) showed that, of 23 subclones which were classified as morphologically transformed either when isolated or when subsequently checked, only 13 were clearly able to form colonies in agar. This lack of correlation may well be a reflection of the apparently changing morphology. Thus, examination of those 24 subclones whose morphology persisted (Table III) revealed that only 1 out of 13 normal subclones compared with 7 out of 11 transformed subclones were clearly positive in agar.

Once again it is clear from Table II that there is no correlation between PE and either morphology or growth in agar. PEs ranged from 0·7% to 67% for those subclones clearly positive in agar.

Of 15 attempts to isolate subclones from MNU-treated and untreated CHMP/E cultures, 9 were successful, and

TABLE IV.—*Properties of Subclones Derived from CHMP/E Cells Treated with* N*-methyl-*N*-nitrosourea (MNU)*

| Subclone | Treatment (μg/ml MNU) | Morphology* At isolation | Morphology* After 5–6 passages | Plating efficiency (%) | Growth* in agar | Agar plating efficiency (%) |
|---|---|---|---|---|---|---|
| A | 50 | N | N | 0·6 | − | |
| B | 50 | N | N | 8·0 | − | |
| C | 50 | T | N | 6·4 | − | |
| D | 50 | T | N | 6·0 | − | |
| E | 100 | N | N | 10·4 | − | |
| F | 100 | T | T | 11·0 | + | 1·8 |
| G | 100 | T | T | 6·7 | + | 0·2 |
| H | 100 | T | N | 1·9 | − | |
| I | 100 | T | N | 1·6 | − | |

* CHMP/E cells had N morphology and did not grow in agar at the time of treatment.

the data relating to morphology, PE and growth in agar are presented in Table IV. Again there is no correlation between PE and other properties, and there is also apparent change in the morphology of some subclones between isolation and test. However, examination of the later assessment of morphology (Table IV) shows an obvious correlation between transformed appearance and growth in soft agar.

### DISCUSSION

A recent paper by Sanford *et al.* (1974) showed that morphological changes which were characteristic of transformation in Syrian hamster cells did not correlate with subsequent tumour production with any degree of predictability. A number of the subclones described in the present study have been injected into immunosuppressed Chinese hamsters to see if they were malignant, and so far 3 tumours have arisen from transformed prostate cells, but none as yet from normal cells. Thus, comparisons between tumourigenicity and morphology, of the type reported by Sanford *et al.* (1974) have not yet been possible. However, good agreement has been reported between tumourigenicity and growth in soft agar (Kirkland and Pick, 1973; Kirkland *et al.*, 1975; Evans and DiPaolo, 1975) or in a comparable methyl cellulose suspension medium (Freedman and Shin, 1974). It would therefore seem justifiable, for the present, to take growth in soft agar as one indicator of malignancy. Using this as a criterion, from the data presented (Tables I–IV) there appears to be great variation between the 3 cell types studied in the predictability of malignant transformation from morphological changes.

Only the subclones isolated from CHMK/H cultures showed stable morphological change correlating with growth in agar (Table I), and transformation of CHMK/H cells would therefore seem a useful means of detecting carcinogens. However, spontaneous transformation is a frequent phenomenon which may confound the interpretation of data obtained from experiments designed to measure the frequency of chemically-induced transformation (Sanford *et al.*, 1974; Kirkland *et al.*, 1975). The high level of spontaneous morphological change seen in CHMK/H cells (Fig. 1) exemplifies this difficulty: treatment with 10 $\mu$g/ml MCA caused only a doubling in transformation compared with control cultures. Clearly these cells are not as useful for the detection of chemical carcinogens as they at first seem.

The level of spontaneous morphological change in liver and prostate cultures was very low (Figs. 2 and 3), and these cells would therefore seem to be of more use for detecting chemical carcinogens. However, as mentioned previously, morphologically transformed liver cells were not easily recognizable and this is presumably why 33% of the liver subclones changed their morphology over 5–6 passages (Tables II and III) and hence the poor correlation between morphology and growth in soft agar.

Morphologically transformed prostate cells were more easily recognizable (Fig. 6) than transformed liver cells, but some isolated subclones still changed their appearance (Table IV). Two possible reasons for this are: (i) the colonies which were isolated by ring cloning were not homogeneous, and normal and transformed cells growing together in the subclone were not distinguishable one from another, or (ii) at the time of isolation, the cells in the subclone had not achieved a stable phenotype. This latter possibility is indicated by the fact that several passages later there was a good correlation between morphology and growth in agar (Table IV). It is interesting to note here that Evans and DiPaolo (1975) state that guinea-pig cells take 4–18 months of culturing after treatment to achieve full expression of the ability to grow in soft agar and to produce tumours.

Whatever the reason for the time-dependent variation in morphology in ostensibly pure subclones, the task of correlating chemically-induced morphological transformation with malignant change becomes very difficult.

The clearest conclusion to be drawn from the results presented here is that, for Chinese hamster cells at least, there is no correlation between PE and other properties of transformed cells such as altered morphology and growth in soft agar. Chinese hamster cells would appear to differ from mouse cells (Frei and Oliver, 1972) in this respect.

Despite the number of reports published on chemically-induced transformation in recent years, discrepancies still arise when attempts are made by other workers to repeat previously reported experiments (Sanford *et al.*, 1974) or when published criteria of transformation are applied to new systems, as indicated by the data presented here. There would seem to be a long way to go before chemically-induced *in vitro* transformation achieves the degree of reliability enjoyed by virus-induced transformation.

I am grateful to Mrs L. A. Taggart for technical assistance and to Dr J. J. Roberts for inspiration and encouragement in preparing this manuscript. The work was supported by the A.K. Fellowship granted by the Institute of Cancer Research, and by grants to this Institute from the Medical Research Council and Cancer Research Campaign.

### REFERENCES

BERWALD, Y. & SACHS, L. (1963) *In Vitro* Cell Transformation with Chemical Carcinogens. *Nature, Lond.*, **200**, 1182.

CHEN, T. T. & HEIDELBERGER, C. (1969*a*) *In Vitro* Malignant Transformation of Cells Derived from Mouse Prostate in the Presence of 3-methylcholanthrene. *J. natn. Cancer Inst.*, **42**, 915.

CHEN, T. T. & HEIDELBERGER, C. (1969*b*) Quantitative Studies on the Malignant Transformation of Mouse Prostate Cells by Carcinogenic Hydrocarbons *in Vitro*. *Int. J. Cancer*, **4**, 166.

DIPAOLO, J. A. & DONOVAN, P. J. (1967) Properties of Syrian Hamster Cells Transformed in the Presence of Carcinogenic Hydrocarbons. *Expl Cell Res.*, **48**, 361.

DIPAOLO, J. A., DONOVAN, P. & NELSON, R. (1969*a*) Quantitative Studies of *in Vitro* Transformation by Chemical Carcinogens. *J. natn. Cancer Inst.*, **42**, 867.

DIPAOLO, J. A., NELSON, R. L. & DONOVAN, P. J. (1969*b*) Sarcoma-producing Cell Lines Derived from Clones Transformed *in Vitro* by Benzo($\alpha$) pyrene. *Science, N.Y.*, **165**, 917.

DIPAOLO, J. A., DONOVAN, P. J. & NELSON, R. L. (1971) *In Vitro* Transformation of Hamster Cells by Polycyclic Hydrocarbons: Factors Affecting the Number of Cells Transformed. *Nature, New Biol.*, **230**, 240.

EVANS, C. H. & DIPAOLO, J. A. (1975) Neoplastic Transformation of Guinea Pig Foetal Cells in Culture Induced by Chemical Carcinogens. *Cancer Res.*, **35**, 1035.

FREEDMAN, V. H. & SHIN, S. (1974) Cellular Tumorigenicity in *Nude* Mice: Correlation with Cell Growth in Semi-solid Medium. *Cell*, **3**, 355.

FREI, J. V. & OLIVER, J. (1972) Early Enhancement of Plating Efficiency of Primary Mouse Embryo Cells by the Carcinogen Methylnitrosourea. *Cancer Res.*, **32**, 2747.

KIRKLAND, D. J. & PICK, C. R. (1973) The Histological Appearance of Tumours Derived from Rat Embryo Cells Transformed *in Vitro* Spontaneously and after Treatment with Nitrosomethylurea. *Br. J. Cancer*, **28**, 440.

KIRKLAND, D. J. & VENITT, S. (1976) Chemical Transformation of Chinese Hamster Cells. II. Appearance of Marker Chromosomes in Transformed Cells. *Br. J. Cancer*, **34**, 145.

KIRKLAND, D. J., HARRIS, R. J. C. & ARMSTRONG, C. A. (1975) Spontaneous and Chemically-induced Transformation of Rat Embryo Cell Cultures. *Br. J. Cancer*, **31**, 329.

MACPHERSON, I. & MONTAGNIER, L. (1964) Agar Suspension Culture for the Selective Assay of Cells Transformed by Polyoma Virus. *Virology*, **23**, 291.

PUCK, T. T., MARCUS, P. I. & CIECIURA, S. J. (1956) Clonal Growth of Mammalian Cells *in Vitro*, Growth Characteristics of Colonies from Single HeLa Cells with and without " Feeder " Layer. *J. exp. Med.*, **103**, 273.

REZNIKOFF, C. A., BRANKOW, D. W. & HEIDELBERGER, C. (1973*a*) Establishment and Characterization of a Cloned Line of C3H Mouse Embryo Cells Sensitive to Postconfluence Inhibition of Division. *Cancer Res.*, **33**, 3231.

REZNIKOFF, C. A., BERTRAM, J. S., BRANKOW, D. W. & HEIDELBERGER, C. (1973*b*) Quantitative and Qualitative Studies of Chemical Transformation of Cloned C3H Mouse Embryo Cells Sensitive to Postconfluence Inhibition of Cell Division. *Cancer Res.*, **33**, 3239.

SANFORD, K. K., HANDLEMAN, S. L., FOX, C. H., BURRIS, J. F., HURSEY, M. L., MITCHELL, J. T., JACKSON, J. L. & PARSHAD, R. (1974) Effects of Chemical Carcinogens on Neoplastic Transformation and Morphology of Cells in Culture. *J. natn. Cancer Inst.*, **53**, 1647.

YERGANIAN, G. & LAVAPPA, K. S. (1971) Procedures for Culturing Diploid Cells and Preparation of Meiotic Chromosomes from Dwarf Species of Hamsters. In *Chemical Mutagens, Principles and Methods for their Detection*, Vol. 2. New York and London: Plenum Press. p. 387.